# Level of hemoglobin among cow milk and camel milk consuming young children: A comparative study

**DOI:** 10.1371/journal.pone.0247572

**Published:** 2021-03-04

**Authors:** Ahmed Abdurahman, Dawd Gashu

**Affiliations:** 1 Department of Food Science and Nutrition, Jigjiga University, Jijiga, Ethiopia; 2 Center for Food Science and Nutrition, Addis Ababa University, Addis Ababa, Ethiopia; Peking University, CHINA

## Abstract

**Background:**

Cow milk is an important source of macro-and micronutrients. However, it has low iron content but high content of casein and calcium thus could negatively influence hemoglobin synthesis. On the other hand, camel milk contains higher iron concentration than cow milk. In addition, the majority of iron in camel milk is associated with the lower molecular fraction of casein suggesting better bioavailability. Furthermore, vitamin C concentration, a useful iron absorption enhancer, is more than three-fold greater in camel milk than cow milk. This study compared hemoglobin concentration among young children consuming consistently cow milk or camel milk.

**Methods:**

Hemoglobin concentration of young children (aged 6–59 mo) from settled pastoralist communities of the Somali region, Ethiopia, consistently consuming cow milk (n = 166) or camel milk (n = 166) was determined. In addition, socio-demographic and water, sanitation, and hygienic (WASH) conditions of study participants’ households were captured. Furthermore, dietary intake and anthropometric characteristics of participating children were assessed.

**Results:**

Among the participating children, 38.6% were underweight, 33.4% were stunted, and 34.5% were wasted. In addition, 77.4% of children were anemic. The present study households had poor WASH conditions. Only 0.6% of children had the minimum acceptable dietary diversity. There was small but significant mean hemoglobin difference among camel milk and cow milk consuming children (9.6±1.8 g/dl vs 9.1±2.2 g/dl; p = 0.012). In addition, the odds of low hemoglobin concentration was greater among cow milk consuming children than camel milk consuming children [AOR 2.17; 95 CI; 1.39, 3.37; p = 0.001]. However, the overall anemia prevalence among the two groups was similar.

**Conclusion:**

Camel milk consumption is associated with better hemoglobin concentration but may not be sufficient to prevent anemia in populations from resource poor settings. The etiology of anemia is multifactorial thus further studies on the link between milk consumption and hemoglobin concentration are important.

## Background

Anemia, low blood hemoglobin concentration, is a major public health concern worldwide affecting about two billion people. Populations from poor resource settings are the most affected. Anemia is associated with higher risk of mortality, low physical and mental development, and compromised productivity in adults. Multiple, often coexisting, factors including consumption of foods with low concentration of or poorly bioavailable micronutrients, parasitic infection, chronic and acute infections, malaria, heavy blood loss, and higher physiological demands for iron at certain life stages for hemoglobin synthesis contribute to low blood hemoglobin concentration [1]. Poor access to clean drinking water, suboptimal sanitation and hygienic facilities are also associated with incidence of anemia through attack by pathogens [2]. In addition, anemia could develop because of hemoglobin disorders (such as sickle cell disease and thalassemias), chronic disease such as AIDS and tuberculosis [3, 4].

Anemia is highly prevalent in populations from Somali and Afar Regions of Ethiopia which are predominantly pastoralist. The Ethiopian Demographic and Health Survey (EDHS) shows that about 83% of children (aged 6–59 mo) and 60% of women in Somali Region are anemic, while the national average prevalence of anemia is 57% in children and 24% in women of reproductive age [5]. Similarly, a study on the spatial distribution of anemia among all regions of Ethiopia using consecutive three time point (2005, 2011, and 2016) EDHS data reported the highest percentage of maternal anemia in Somali region [6]. Cow milk is an important source of key nutrients. However, several available studies indicated that cow milk consumption could be a risk factor for low hemoglobin concentration in young children [7, 8]. A study in Brazil also reported a positive association between milk consumption and anemia prevalence in infants [9]. This is mainly attributed to the low iron content but excess amount of casein and calcium in cow milk. Casein in milk chelates iron by binding with phosphoserine residues preventing the release of iron in a free form consequently impairing intestinal absorption [10]. In addition, calcium inhibits heme and non-heme iron absorption in a dose-dependent manner [11].

On the other hand, camel milk contains greater iron concentration than cow milk (1.35–2.5mg/L vs 0.3–0.8mg/L) [12]. In addition, the majority of iron is associated with the lower molecular fraction of casein suggesting better bioavailability to increase iron store and hemoglobin synthesis [13]. Furthermore, vitamin C concentration is more than three-fold in camel milk than cow milk [14]. Vitamin C inherent to the food or added from external sources enhance iron absorption from non-heme sources by reducing ferric iron into the readily bioavailable form- ferrous iron. Vitamin C can also overcome the iron absorption inhibition effect due to high calcium and proteins in milk [15]. These characteristics may indicate that camel milk consumption could be more beneficial for better hemoglobin synthesis to consumers than cow milk. Therefore, the present study aimed to assess and compare hemoglobin concentration among young children (aged 6–59 mo) consistently and predominantly consuming cow milk and camel milk from pastoralist communities of the Somali Region in Ethiopia.

## Materials and methods

### Study location description

The study was conducted in four administrative zones (Shabele, Fafan, Jarar, Erer) of Somali regional state located at east and southeast of Ethiopia. The region is known for high incidence of energy and micronutrient undernutrition. A recent study reported 75% of children at the age of 1–5 years are anemic and 91% had iron deficiency [16]. In addition, 31.7%, 30.5% and 21.1% of under-five children are underweight, stunted and wasted [17]. Slightly more than one third of the inhabitants in the region are pastoralists. The four zones were selected purposefully because significant portion of inhabitants livelihood depend on livestock production thus milk consumption is very common. Similarly, one *woreda* from each zone where we can find pastoral livestock populations rearing cattle (but not camel) and camel (but not cattle) was selected. Furthermore, one district from each *woreda* was randomly selected.

In the selected districts, a house-to-house census was conducted to register all children aged 6–59 mo. A total of 332 children of 6–59 mo old (166 children from camel rearing households thus consuming consistently and exclusively camel milk and another 166 children of households consistently and exclusively consuming cow milk) were selected randomly based on the sample size calculation considering 83% prevalence of any form of anemia in children 6–59 mo [5], a 95% confidence level and 1.5 design effect.

### Data collection

Recruited data collectors were trained to standardized methods of administering interview questions, and conducting anthropometric measurement. A structured questionnaire was prepared in the local language and pretested outside the study districts before applying to the real data collection. The questionnaire was used to capture socio-demographic information and WASH conditions of the study households and dietary intake of participating children. The study was conducted between December 2017-February 2018.

### Dietary intake assessment

Dietary intake was assessed by individual 24 hr recall method. Mothers or caretakers were asked to report the type of food and drinks the child consumed 24 hrs preceding the survey. The food items consumed by participating children were further categorized into food groups to calculate their dietary diversity score according to the FAO guideline to assess individual dietary diversity score [18].

### Water, sanitation and hygiene practices

Respondents were asked the source of drinking water for the study households. In addition information regarding type of toilet and waste disposal practice of the households were collected by interview and/or observation. Drinking water sources and sanitation facilities of the study households were categorized as improved or unimproved based on the WHO/UNICEF joint monitoring program for water supply, sanitation and hygiene guideline [19]. Morbidity status of children was assessed by asking caretakers whether children had an episode of diarrhea (passing watery stools three or more times per day), fever (fever with no other symptoms) or acute respiratory infection in the last 14 days preceding the survey.

### Anthropometry

Anthropometry measurers were trained on weight and height measurement of young children before the survey. The main measurers were evaluated and passed the standardization exercise for measurement precision and accuracy [20]. Weight of children was measured using a digital scale (T 128 ANITA WB-100; Tanita Corporation, Arlington Heights, IL, USA) while children wore light clothes and were barefoot. The scale was tared to read zero before starting the measurements. The weight scale was calibrated at least once a day using an object of known weight. Length/height was measured using a calibrated wooden height measuring board (Stadiometer; Shorr Productions, Olney, MD, USA) while children were barefoot. The weight reading was recorded twice and length/height was measured three times and the average of the measurement was used for analysis. The measurements were converted into z-scores of weight-for-age (WAZ), length/height-for-age (LAZ/HAZ) and weight-for-height (WHZ) using WHO Anthro software to determine the nutritional status of participating children.

### Hemoglobin measurement

Experienced nurses measured the hemoglobin level of children onsite using a digital photometer (HemoCue, Hb 201 DM; Ängelholm, Sweden) by pricking the fingertip with sterile disposal lancet after cleaning with alcohol wipe. The finger was squeezed to get a drop of blood. The altitude of the study districts was measured using Garmin GPS (Garmin GPS 72H, Taiwan). The hemoglobin concentration was adjusted for altitude using the formula by Sullivan et al. [21]. Children with Hb concentrations in whole blood<11.0 g/dl were considered anemic and graded as mildly anemic (10.0–10.9 g/dl), moderately anemic (7–9.9 g/dl) and severely anemic (<7 g/dl).

### Statistical analysis

Statistical analysis of data was performed using the statistical soft-ware package PASW Statistics for Window (v18, Chicago, IL, USA). Descriptive statistics were used to present results. Normal distribution of data was checked with the Kolmogorov–Smirnov test. Bivariate correlations were computed using Pearson correlation test. Variables such as age, family size, dietary diversity score, and volume of milk consumed, hemoglobin concentration, and weight and height among camel milk and cow milk consuming children were compared using Student’s t-test. Chi-square test was used to compare proportions of outcomes between two independent groups. The relationship between anemia status (as a dichotomous variable) and potential predictors was tested using bivariate and multivariable binary logistic regression.

### Ethical approval and consent to participate

All procedures involving human subjects were approved by Institutional Review Board of Addis

Ababa University, College of Natural Sciences, CNSDO/318/11/2018. A support letter was also obtained from the Somali Region Health Bureau. Written informed consent was obtained from parents or guardians of children. Numerical codes were assigned to all study participant for anonymity.

## Results

The study children were from areas of similar altitude. The study households (n = 332) had 2 to 12 family size, with a mean value 5.6±2.4. All study participants were from the rural areas and were Somali in ethnicity. The study children (n = 332) were in the age of 6–59 mo with mean value 33.3±17.2 mo. In both cases, majority of households had unimproved sources of drinking water and toilet facilities. Only a child among the study participants experienced sickness within two weeks preceding the survey.

The frequency of food group consumption among the present study children was greatest for milk (100%), cereals (86.7%), and other vegetables (61.7%). Few or none of the children consumed eggs, meat and fish, organ meat, vitamin A rich fruits, dark green leafy vegetables, vitamin A rich vegetables, or other fruits and vegetables (Table 1). Both camel milk and cow milk consuming children had in general low dietary diversity score. Same percentage (99.4%) of children in both groups had lower than the minimum acceptable dietary diversity (< 4 food groups) 24 hr preceding the interview. Yet, cow milk consuming children had significantly higher dietary diversity score compared to camel milk consuming children (3.1±1.0 vs 2.7±1.0; p = 0.001).

**Table 1:** Percentage distribution of intake of different food items in the 24 h preceding the survey by young children from pastoralist communities of the Somali Region, Ethiopia, 2018.

Food item	Camel milk consuming (n = 166), % (n)	Cow milk consuming (n = 166), % (n)
Maize	51.5 (85)	78.4 (131)
Rice	27.3 (45)	12.0 (20)
Wheat	60.0 (99)	68.3 (114)
Sorghum	35.2 (58)	79.6 (133)
Potato	4.8 (8)	4.8 (8)
Onion	58.8 (97)	64.7 (108)
Banana	0.6 (1)	0.6 (1)
Tomato	24.8 (41)	52.7 (88)
lemon	0.6 (1)	0.0 (0)
Pineapple	1.2 (2)	0.0 (0)
Peas	1.2 (2)	0.0 (0)
Milk	100 (166)	100 (166)

In the present study, all the three forms of undernutrition were prevalent affecting almost similar percent of camel milk (37% underweight, 35.6% stunting, and 33.1% wasting) and cow milk (40.2% underweight, 32% stunting and 37.3% wasting) consuming children (Table 2).

**Table 2:** Nutritional status of young children from pastoralist communities of the Somali Region, Ethiopia, 2018.

Variables		Camel milk consuming (n = 166), % (n)	Cow milk consuming (n = 166), % (n)
Underweight			
	Overall*	37.0 (61)	40.2 (57)
	Moderate†	18.2 (30)	16.8 (28)
	Severe‡	18.8 (31)	23.4 (29)
Stunting			
	Overall	35.6 (59)	32.0 (53)
	Moderate	17.5 (29)	13.9 (23)
	Severe	18.1 (30)	18.1 (30)
Wasting			
	Overall	33.1 (55)	37.3 (62)
	Moderate	15.6 (26)	15.7 (26)
	Severe	17.5 (29)	21.6 (36)

*Overall: z-score < -2

^†^Moderate: Z score between -2 and -3

^‡^Severe: z-score < -3.

Children had hemoglobin value in the range of 3.2–13.9 g/dl with mean value of 9.2±2.0 g/dl. In general, 77.5% of children were anemic. Mild anemia was present in 17.2% of children, 45.5% had moderate anemia, and 14.8% had severe anemia. There was small but significant (p = 0.012) difference in hemoglobin concentration among camel milk consuming children (9.6±1.8g/dl) compared to cow milk consuming children (9.1±2.2g/dl). However, the proportion of anemia in both camel milk and cow milk consuming children was similar. On the other hand, greater prevalence of severe anemia was evident in camel milk consuming children while the prevalence moderate anemia was greater in camel milk consuming children (Table 3).

**Table 3:** Prevalence of anemia among camel milk and cow milk consuming young children from the Somali Region, Ethiopia, 2018.

	Camel milk, n (%)	Cow milk, n (%)	Chi-square	p-value
Mild anemia (10–10.9 g/dl)	27 (16.3)	30 (18.1)	0.19	0.66
Moderate anemia (7.0–9.9g/dl)	85 (51.2)	66 (39.8)	4.38	0.03
Severe anemia (<7.0 g/dl)	18 (10.8)	31 (18.7)	4.04	0.04

The present study children had almost similar household and individual characteristics. However, significantly higher proportion of households of cow milk consuming children defecate outside in nature (X^2^(1) = 3.92; p = 0.048), and dispose household wastes improperly (X^2^(1) = 26.33, p<0.001). On the other hand, significantly higher percentage of households of camel milk consuming children use unprotected dug well as a source of drinking water (X^2^(1) = 15.77; p<0.001) than boreholes. In addition, cow milk consuming children had significantly higher dietary diversity score compared to camel milk consuming children (Table 4). However, dietary diversity score in the present study was not significantly associated with hemoglobin concentration of both camel milk [95% CI 0.54; 0.25, 1.15; p = 0.11] and cow milk [95% CI 0.86; 0.43, 1.74; p = 0.69] consuming children.

**Table 4:** Comparison of factors among cow milk and camel milk consuming young children from pastoralist communities of the Somali Region, Ethiopia, 2018.

		Camel milk consuming (n = 166),range, mean ± sd	Cow milk consuming (n = 166),range, mean ± sd	p-value
Age (mo)		6–59, 33.2±17.3	6–59, 33.4±17.0	0.91
Sex				
	female	59.4 (98)	53.3 (89)	0.639
	male	40.6 (67)	46.7 (78)	
Breast milk	yes	21.2 (35)^†^	23.4 (39)	0.598
Source of drinking water				
	dug well	98.8 (164)	88.0 (146)	<0.001
	bore hole	1.2 (2)	12.0 (20)	
Household waste disposal				
	burn or bury	50.3(84)	22.8(38)	<0.001
	dispose everywhere	49.1(82)	77.2(128)	
Toilet facility				
	outdoors in nature	93.9(156)	98.2(163)	<0.001
	pit latrine	6.1(10)	1.8(3)	
Family size		3–12, 5.8±2.5	3–12, 5.3±2.3	0.06
Number of camels		1–44, 6 [2,10]^‡^	0–2, 0.01±0.5	<0.001
Number of cows		0–2, 0.03±0.9	1–40, 2 [2,5]	<0.001
Volume of milk consumed (ml)		50–1800; 420.8±270.2	20–1150; 418.6±207	0.97
Weight of children (kg)		4–22; 11.3±3.6	3–22, 11.2±4.2	0.82
Height of children (cm)		50–115, 87.2±14.9	58–124, 87.6±15.8	0.83
WAZ		-5.7–4.2, -1.2 [-2.6,-0.3]	-6.8–3.0; -1.4 [-2.9,-0.07]	0.14
HAZ		-6.2–4.7, -1.2 [-2.3,-0.2]	-6.6–4.6, -1.2 [-2.4,-0.07]	0.52
WHZ		-5.6–4.5, -0.9 [-2.3, 0.2]	-6.3–5.0, -1.4 [-2.5,-0.3]	0.18
Dietary diversity		1–4, 2.7±1.0	1–5, 3.1±1.0	0.001

^†^All such values are % (n)

^‡^such expressions are median [first quartile, third quartile].

Several potential factors including breast milk consumption, source of drinking water, type of disposal of household wastes, and place of defecation that influence hemoglobin concentration in addition to the type of milk consumed by the children were tested in logistic regression. Household waste disposal and type of milk consumption were significantly associated with occurrence of anemia. Children from households that dispose household wastes improperly everywhere had 2.1[95% CI; 1.11; 4.07; p<0.02] times increased odds of anemia in the bivariate binary logistic regression. However, the association was not significant (p = 0.512) when other factors were considered in the multivariate logistic regression model (Table 5). In the multivariate analysis, the odds of low hemoglobin concentration was greater among cow milk consuming children than camel milk consuming children [AOR 2.17; 95 CI; 1.39, 3.37; p = 0.001].

**Table 5:** Association between child and household characteristics and low hemoglobin among young pastoralist children in the Somali Region, Ethiopia, 2018.

Variables		n	AOR [95%CI]	p-value
The child is breast-fed^†^				
	No	17		
	Yes	74	1.3 [0.85, 1.99]	0.21
Source of drinking water for the household				
	Dug well	310		
	Bore hole	22	1.4 [0.54, 2.36]	0.47
Type of milk the child consumes				
	Camel milk	166		
	Cow milk	166	2.17 [1.39,3.37]	0.001
Household waste disposal				
	Burn or bury	122		
	Dispose everywhere	210	1.14 [0.76,1.73]	0.43
Toilet facility				
	Outdoors in nature	319		
	Pit latrine	13	1.55 [0.40, 5.99]	0.52

^†^Only children 6–24 mo old were considered.

## Discussion

The present study compared hemoglobin concentration among camel milk and cow milk consuming young children from resource poor pastoral communities of the Somali region, Ethiopia. In general, 77.4% of the children had low hemoglobin concentration suggesting that anemia is a severe public health problem in the area. Compared to cow milk consuming children, those consuming camel milk had slightly greater mean hemoglobin concentration. However, the prevalence of anemia in both groups was similar. Controlling for other individual and household characteristics, the odds of low hemoglobin concentration was greater among cow milk consuming children than camel milk consuming children [AOR 2.17; 95 CI; 1.39, 3.37; p = 0.001].

Diversified diets are important to provide adequate intake of vitamins and minerals. However, communities from resource poor settings are commonly affected by the deficiency of one or more micronutrients mainly because their diet lacks diversification. Almost all (99.4%) of the children in the present study had less than the minimum acceptable dietary diversity score. Yet, compared to camel milk consuming children, cow milk consuming children had significantly higher mean dietary diversity score. However, dietary diversity in the present study children was not associated with hemoglobin concentration. Meat, fish, and organ meats are rich in the bioavailable form of iron. In addition, these animal foods are good source of high-quality proteins with the capacity to enhance iron absorption from non-heme sources consequently increasing blood hemoglobin concentrations [22–24]. Even though pastoralist communities possess livestock and are known to rely on animal source foods for energy and micronutrient intake [25], none of the present study children consumed eggs, meat and fish, and organ meat 24 hr preceding the survey. However, a single 24-hour recall method of dietary assessment suffers from recall bias and may not represent long term dietary patterns of the study participants [26].

All three forms of undernutrition (underweight, stunting and wasting) were highly prevalent in both groups of the present study children. Similarly, the 2019 mini Ethiopian Demographic and Health Survey (EDHS) report shows that in the Somali region 31.7%, 30.5%, 21.1% of under five children are underweight, stunted and wasted while the national average for underweight is 21%, 7% wasting and 37% stunting. The Somali region has the highest wasting and underweight prevalence [17]. Diarrheal disease, inappropriate breast feeding, and low dietary diversification are the most common risk factors of child undernutrition in Somali [27].

The present study children have almost homogenous individual and household characteristics except that higher proportion of households of cow milk consuming children defecate outside in nature and dispose household wastes improperly. On the other hand, significantly higher percentage of households of camel milk consuming children use unimproved sources of drinking water. Lack of safe drinking water, poor hygiene and sanitary facilities expose children to attack by pathogens causing infection that affect children’s capacity to respond to nutrients and exacerbate nutrient losses through diarrhea and vomiting. This consequently causes anemia in young children [28]. A study reported lack of sanitation access as a significant risk factor for anemia in children [29]. On the other hand, WASH interventions were effective to reducing the risk of anemia in Kenyan and Bangladeshi children [30]. In the present study, children from households that dispose household wastes improperly had higher odds of anemia in the bivariate binary logistic regression; however, the association was not significant when other factors were considered.

Milk is a concentrated source of macro- and micronutrients important for promoting physical and cognitive development of children. In addition, it reduces child morbidity and mortality [31]. However, cow milk consumption may exposes infant and toddlers to the risk of iron store depletion and low hemoglobin concentration. Several mechanisms linking cow milk consumption with iron deficiency and anemia have been identified. The inherent low iron content of cow milk, inducing intestinal bleeding in infants, and iron absorption inhibition effect of calcium and casein in cow milk from non-heme sources are among the most important mechanism [32]. On the other hand, compared to cow milk, camel milk has higher iron and vitamin C concentration but lower casein that favor iron store enhancement thus hemoglobin synthesis [11, 13]. In the present study, the mean hemoglobin concentration in camel milk consuming children was significantly higher than cow milk consuming children. However the overall percentage of anemia in cow milk and camel milk consuming children was similar. This may suggest the benefit of consuming camel milk over cow milk for better hemoglobin concentration but not sufficient enough to ensure normal hemoglobin concentrations may be due to the effect of other contributing factors.

It is difficult to link solely milk consumption and hemoglobin concentration in epidemiological studies because several factors directly or indirectly affect the hemoglobin synthesis process which were not captured in the present study including socio-economic status and household food insecurity [33], presence of chronic disease [34], and parasitic infections [35, 36]. The deficiency of vitamins and mineral also contribute to low hemoglobin concentration. For example, iron deficiency is assumed to account up to 37% all causes of anemia [37]. In addition, the deficiency of other micronutrients such as zinc, vitamin A, folate, riboflavin are notable causes of anemia [36].

## Conclusion

In the present study, there was small but significant mean hemoglobin difference among camel milk and cow milk consuming children. However, the overall anemia prevalence was similar in both groups. This may indicate the importance of camel milk to increasing hemoglobin concentration compared to cow milk in young children but not sufficient to overcome the occurrence of anemia. This is because the etiology of low hemoglobin concentration is multifaceted especially in populations from resource poor settings thus further studies that show cause effect relationship to see clear effect of camel milk on hemoglobin concentration are important.

## Supporting information

S1 File (SAV)

## References

[1] De Benoist B, Cogswell M, Egli I, McLean E. Worldwide prevalence of anaemia 1993–2005; WHO Global Database of anaemia. 2008. https://stacks.cdc.gov/view/cdc/5351. Accessed 16 January, 2020. 10.1017/S1368980008002401 18498676

[2] Kothari MT, Coile A, Huestis A, Pullum T, Garrett D, Engmann, C. Exploring associations between water, sanitation, and anemia through 47 nationally representative demographic and health surveys. Ann. N. Y. Acad. Sci. 2019; 1450: 249–267. 10.1111/nyas.14109 31232465 PMC6771505

[3] Hella J, Cercamondi CI, Mhimbira F, Sasamalo M, Stoffel N, Zwahlen M, et al. Anemia in tuberculosis cases and household controls from Tanzania: contribution of disease, coinfections, and the role of hepcidin. PloS one. 2018; 13: e0195985. 10.1371/journal.pone.0195985 29677205 PMC5909902

[4] Chaparro CM, Suchdev PS. Anemia epidemiology, pathophysiology, and etiology in low-and middle-income countries. Ann N Y Acad Sci. 2019; 1450, 15–31. 10.1111/nyas.14092 31008520 PMC6697587

[5] Central Statistical Agency (CSA) [Ethiopia] and ICF. Ethiopia Demographic and Health Survey. Addis Ababa, Ethiopia, and Rockville, Maryland, USA: CSA and ICF. 2016. https://dhsprogram.com/pubs/pdf/FR328/FR328.pdf. Accessed 27 November, 2019.

[6] Liyew AM, Kebede SA, Agegnehu CD, Teshale AB, Alem AZ, Yeshaw Y, et al. Spatiotemporal patterns of anemia among lactating mothers in Ethiopia using data from Ethiopian Demographic and Health Surveys (2005, 2011 and 2016). PloS one. 2020; 15: e0237147. 10.1371/journal.pone.0237147 32760116 PMC7410320

[7] Oliveira MA, Osório MM. Cow’s milk consumption and iron deficiency anemia in children. Jornal de pediatria. 2005; 81: 361–367. 10.2223/JPED.1386 16247536

[8] Elalfy MS, Hamdy AM, Maksoud SSA, Megeed RIA. Pattern of milk feeding and family size as risk factors for iron deficiency anemia among poor Egyptian infants 6 to 24 months old. Nutr Res. 2012; 3: 93–99. 10.1016/j.nutres.2011.12.017 22348457

[9] Hadler MCC, Colugnat FA, Sigulem DM. Risks of anemia in infants according to dietary iron density and weight gain rate. Prev Med. 2004; 39: 713–721. 10.1016/j.ypmed.2004.02.040 15351537

[10] Kibangou IB, Bouhallab S, Henry G, Bureau F, Allouche S, Blais A, et al. Milk proteins and iron absorption: contrasting effects of different caseinophosphopeptides. Pediatr Res. 2005; 58: 731–734. 10.1203/01.PDR.0000180555.27710.46 16189201

[11] Roughead ZK, Zito CA, Hunt JR. Inhibitory effects of dietary calcium on the initial uptake and subsequent retention of heme and nonheme iron in humans: comparisons using an intestinal lavage method. Am J Clin Nutr. 2005; 82: 589–597. 10.1093/ajcn.82.3.589 16155272

[12] Wernery U. Camel milk, the white gold of the desert. J. Camel Pract. Res. 2006; 13: 15–26.

[13] Al-Awadi FM, Srikumar TS. Trace elements and their distribution in protein fractions of camel milk in comparison to other commonly consumed milks. J Dairy Res. 2001; 68: 463–469. 10.1017/s0022029901005003 11694048

[14] Yadav AK, Kumar R, Priyadarshini L, Singh J. Composition and medicinal properties of camel milk: A Review. Asian J Dairy Food Res. 2015; 34: 83–91.

[15] Hurrell R, Egli I. Iron bioavailability and dietary reference values. Am J Clin Nutr. 2010; 91: S1461–S1467. 10.3945/ajcn.2010.28674F 20200263

[16] Osman KA, Zinsstag J, Tschopp R, Schelling E, Hattendorf J, Umer A, et al. Nutritional status and intestinal parasites among young children from pastoralist communities of the Ethiopian Somali region. Matern Child Nutr. 2020;16: e12955. 10.1111/mcn.12955 32026575 PMC7296781

[17] EPHI, ICF. Ethiopia Mini Demographic and Health Survey: Key Indicators. Rockville, Maryland, USA: EPHI and ICF. 2019. https://dhsprogram.com/pubs/pdf/PR120/PR120.pdf. Accessed 20 October, 2019.

[18] Kennedy G, Ballard T, Dop MC. Guidelines for measuring household and individual dietary diversity. Food and Agriculture Organization of the United Nations. 2011. http://www.fao.org/3/a-i1983e.pdf. Accessed 15 January, 2020.

[19] WHO and UNICEF. Joint monitoring programme for water supply and sanitation. Global water supply and sanitation assessment 2000 report. Washington, DC: WHO/UNICEF. 2000. https://www.who.int/water_sanitation_health/monitoring/jmp2000.pdf. Accessed 15 January, 2020.

[20] WHO and UNICEF. Recommendations for data collection, analysis and reporting on anthropometric indicators in children under 5 years old. Geneva. 2019. https://apps.who.int/iris/bitstream/handle/10665/324791/9789241515559-eng.pdf. Accessed 11 December, 2020.

[21] Sullivan KM, Mei Z, Grummer‐Strawn L, Parvanta I. Haemoglobin adjustments to define anaemia. Trop Med Int Health. 2008; 13: 1267–1271. 10.1111/j.1365-3156.2008.02143.x 18721184

[22] Hurrell RF, Reddy MB, Juillerat M, Cook JD. Meat protein fractions enhance nonheme iron absorption in humans. Nutr J. 2006; 136: 2808–2812. 10.1093/jn/136.11.2808 17056805

[23] Reddy MB, Hurrell RF, Cook JD. Meat consumption in a varied diet marginally influences nonheme iron absorption in normal individuals. Nutr J. 2006; 136: 576–581. 10.1093/jn/136.3.576 16484527

[24] Neumann C, Harris DM, Rogers LM. Contribution of animal source foods in improving diet quality and function in children in the developing world. Nutr Res. 2002; 22: 193–220.

[25] Iannotti L, Lesorogol C. Dietary intakes and micronutrient adequacy related to the changing livelihoods of two pastoralist communities in Samburu, Kenya. Curr Anthropol. 2014; 55: 475–482.

[26] Gibson RS. Principles of Nutritional Assessment. 2nd ed. New York: Oxford University Press, Inc; 2005.

[27] Fekadu Y, Mesfin A, Haile D, Stoecker BJ. Factors associated with nutritional status of infants and young children in Somali Region, Ethiopia: a cross-sectional study. BMC Public health. 2015; 15: 846. 10.1186/s12889-015-2190-7 26330081 PMC4557759

[28] Budge S, Parker AH, Hutchings PT, Garbutt C. Environmental enteric dysfunction and child stunting. Nutr Rev. 2019; 77: 240–253. 10.1093/nutrit/nuy068 30753710 PMC6394759

[29] Larsen DA, Grisham T, Slawsky E, Narine L. An individual-level meta-analysis assessing the impact of community-level sanitation access on child stunting, anemia, and diarrhea: Evidence from DHS and MICS surveys. PLoS Negl Trop Dis. 2017; 11: e0005591. 10.1371/journal.pntd.0005591 28594828 PMC5464528

[30] Stewart CP, Dewey KG, Lin A, Pickering AJ, Byrd KA, Jannat K, et al. Effects of lipid based nutrient supplements and infant and young child feeding counseling with or without improved water, sanitation, and hygiene (WASH) on anemia and micronutrient status: results from cluster-randomized trials in Kenya and Bangladesh. Am J Clin Nutr. 2019; 109: 148–164. 10.1093/ajcn/nqy239 30624600 PMC6358037

[31] Dror DK, Allen LH. The importance of milk and other animal-source foods for children inlow-income countries. Food Nutr Bull. 2011; 32: 227–243. 10.1177/156482651103200307 22073797

[32] Ziegler EE. Consumption of cow’s milk as a cause of iron deficiency in infants and toddlers. Nutr Rev. 2011; 69: S37–S42. 10.1111/j.1753-4887.2011.00431.x 22043881

[33] Pasricha SR, Black J, Muthayya S, Shet A, Bhat V, Nagaraj S, et al. Determinants of anemia among young children in rural India. Pediatrics. 2010; 126: e140–9. 10.1542/peds.2009-3108 20547647

[34] Koshy SM, Geary DF. Anemia in children with chronic kidney disease. Pediatr Nephrol. 2008; 23: 209–219. 10.1007/s00467-006-0381-2 17245602 PMC2668634

[35] Koc A, Kösecik M, Vural H, Erel O, Ataş A, Tatli MM. The frequency and etiology of anemia among children 6–16 years of age in the southeast region of Turkey. Turk J Pediatr. 2000; 42: 91–95. 10936971

[36] Righetti AA, Koua AYG, Adiossan LG, Glinz D, Hurrell RF, N’Goran EK, et al. Etiology of anemia among infants, school-aged children, and young non-pregnant women in different settings of south-central Côte d’Ivoire. Am J Trop Med Hyg. 2012; 87: 425–434. 10.4269/ajtmh.2012.11-0788 22848097 PMC3435343

[37] Petry N, Olofin I, Hurrell R, Boy E, Wirth J, Moursi M, et al. The proportion of anemia associated with iron deficiency in low, medium, and high human development index countries: a systematic analysis of national surveys. Nutrients. 2016; 8: 693. 10.3390/nu8110693 PMC5133080 27827838

